# Optimal selection of epitopes for TXP-immunoaffinity mass spectrometry

**DOI:** 10.1186/1748-7188-5-28

**Published:** 2010-06-25

**Authors:** Hannes Planatscher, Jochen Supper, Oliver Poetz, Dieter Stoll, Thomas Joos, Markus F Templin, Andreas Zell

**Affiliations:** 1 University of Tübingen, Center for Bioinformatics, Sand 1, D-72076 Tübingen, Germany; 2 Natural and Medical Science Institute at the University of Tübingen, Markwiesenstraße 55, D-72770 Reutlingen, Germany; 3 University of Applied Sciences, Albstadt-Sigmaringen, Anton-Günther-Str 51, D-72488 Sigmaringen, Germany; 4 Current address: Genomatix Software GmbH, Bayerstr. 85a, 80335 Munich, Germany

## Abstract

**Background:**

Mass spectrometry (MS) based protein profiling has become one of the key technologies in biomedical research and biomarker discovery. One bottleneck in MS-based protein analysis is sample preparation and an efficient fractionation step to reduce the complexity of the biological samples, which are too complex to be analyzed directly with MS. Sample preparation strategies that reduce the complexity of tryptic digests by using immunoaffinity based methods have shown to lead to a substantial increase in throughput and sensitivity in the proteomic mass spectrometry approach. The limitation of using such immunoaffinity-based approaches is the availability of the appropriate peptide specific capture antibodies. Recent developments in these approaches, where subsets of peptides with short identical terminal sequences can be enriched using antibodies directed against short terminal epitopes, promise a significant gain in efficiency.

**Results:**

We show that the minimal set of terminal epitopes for the coverage of a target protein list can be found by the formulation as a set cover problem, preceded by a filtering pipeline for the exclusion of peptides and target epitopes with undesirable properties.

**Conclusions:**

For small datasets (a few hundred proteins) it is possible to solve the problem to optimality with moderate computational effort using commercial or free solvers. Larger datasets, like full proteomes require the use of heuristics.

## Background

Mass spectrometry (MS) based protein profiling has become one of the key technologies in biomedical research and biomarker discovery. Contrary to the analysis of mRNA profiles, the screening of protein expression profiles allows direct conclusions about the molecular mechanisms involved in a certain condition, because many cellular processes are directly related to the protein functions.

mRNA-Profiling is based on hybridization of DNA-molecules and binding molecules are easy to postulate and to synthesize. This allows the comparatively cheap production of high-density microarrays that cover a large portion of the known genome. Unfortunately this is not applicable in the protein world since features of protein binding molecules can not be predicted as easily.

Mass spectrometry allows a parallel, high-throughput detection of a mixture containing a limited number of peptides [1-3]. For qualitative and quantitative protein profiling of a complex sample time-consuming sample fractionation steps such as 2D gel electrophoresis or multidimensional chromatography are necessary. In this way, small subsets of the sample are analyzed fraction by fraction. The mentioned fractionation methods are the limiting factor in MS-based protein analysis.

Immunoaffinity-MS approaches combine antibody-based approaches with mass-spectrometry, increasing sample throughput and detection sensitivity by capturing proteins or peptides from the sample using protein-or peptide-specific antibodies [4-9]. However, the drawback is the large number of antibodies needed - one antibody per protein. Nevertheless efforts are ongoing to generate antibodies for the analysis of the plasma proteome by an immunoaffinity MS approach [10].

**Figure 1:**
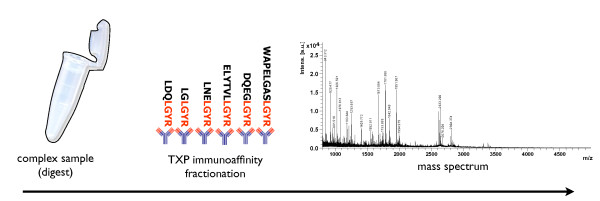
**Schematic Immunoaffinity-MS workflow: Sample preparation and digest, fractionation with TXP-antibodies, analysis of the fraction with mass spectrometry**.

The novel 'Triple X proteomics'-strategy (TXP) [11] uses a special kind of antibodies to immunoprecipitate groups of peptides which share a common short sequence (3-5 amino acids) at the N-or C-terminal end, generated in a tryptic whole proteome digest of a biological sample (see Figure 1). In contrast to classical peptide antibodies those binders can be selected and generated to bind dozens to hundreds of peptides sharing the same TXP-epitope.

As the biological proof of concept has been shown, the practical question arose which epitopes should be produced to cover a large set proteins with minimal effort based on prior knowledge of a proteome.

In this work we present a method to select and optimize TXP-antigens, the short common terminal sequences (epitopes), to cover a given set of target proteins. This leads to a substantial reduction of antibodies to be generated for a proteome wide immunoaffinity-MS approach. An in-silico digest of a fully elucidated target proteome is filtered to eliminate those peptides with undesirable properties or epitopes. We show that the problem of selecting the minimal set of TXP-antigens is equivalent to the set cover problem. We apply a greedy algorithm and a boolean programming approach, and extend those methods, to enhance the multiple coverage of the protein targets for a better experimental design.

## Methods

The goal of the experiment design task is to calculate a minimal set of epitopes to measure a given set of proteins in a complex mixture. The mixture is a digest, that was derived from a tryptic digest of the whole proteome. It is also assumed that the digest is complete (there are no missed or mis-cleavages) and that the proteome of the organism is fully elucidated. Another assumption is that the hypothetical antibody is specific to a given epitope, and does not bind variations or modifications of the epitope.

The process is divided in a filtering pipeline, where the search space is reduced, and the optimization step, where the problem is formulated and then solved.

### Filter Pipeline

Starting from a proteome dataset (e.g. Uniprot or IPI) that is defined as the background, an in-silico tryptic digest is obtained. It is assumed that the background dataset holds information about all proteins found in the future sample.

Peptides must have certain properties to be detectable by a read-out method. The mass of the peptide has to be known and, in addition, mass-spectrometers have limits in resolution and mass range. Instead of including these limitations in optimization-constraints, a filter pipeline is applied where peptides and epitopes, which do not match the criteria, are removed.

Here, the digest of a proteome *P *is defined by a set of pairs *D*(*P*) = {(*P*_*i*_, *p*_*j*_)} where *p*_*j *_is the j-th peptide in protein *P*_*i*_, *p*_*i *_= *a*_*1*_*a*_*2 *_... *a*_*n *_is an amino acid sequence composed of the single letter amino acid code. We define a peptide-antibody-combination as a quadruple labelled :(1)

Here, *l *defines the length of the epitope and *t *describes whether the terminus is n- or c-terminal. The set(2)

with(3)(4)

contains all combinations for a given proteome, length range and termini. This combination set is the raw start input for the filter pipeline. The quadruple is not needed for every filter, but for reasons of formal continuity we use the definition through the whole specification of the pipeline.

Knowing the weight of captured peptides is essential for the mass spectrometry read-out. Therefore, the *'unknown-positions'-filter *removes peptides containing unknown positions (symbol *X *), as their weight cannot be calculated.

The *methionine filter *removes combinations with epitopes containing methionine (symbol *M *), since chemical modifications of methionine may hamper the recognition of the target epitope by a binding molecule, especially by an antibody.

The *high abundant epitope filter *removes combinations with epitopes which would capture a large number of peptides. An antibody affine to such an epitope would be cluttered, and therefore be rather insensitive.

We define a subset *C*^*e *^⊂ *C *which contains all combinations  where *epitope *. If |*C*^*e*^| is bigger than 600, the epitope *e *would not be considered for optimization.

The *weight filter *removes combinations which share the same terminus and have almost the same weight.

These peptides can not be measured with standard mass spectrometry read-out, because the resulting peaks would overlap in the spectrum. A reasonable value for Δ_*min *_is 2-10 Da for MALDI-TOF-spectrometers. In this filter, rather than excluding the terminus from the optimization only the almost isobaric peptides are not counted as identifiable by combining the specific epitope and mass information. For example the peptides AYEQLGYR and HLEILGYR could not be discriminated in a mass spectrum of a probe enriched with an antibody affine to the epitope LGYR, because the masses only differ by 1.068 Da, if the resolution of the mass spectrometer does not provide the adequate resolution.

The *length filter *removes combinations which do not fit in the detection range of the mass spectrometer. The detection range depends on the technical specifications of the mass-spectrometer, but a range from 8-30 amino acids is a good rule of thumb.

**Figure 2:**
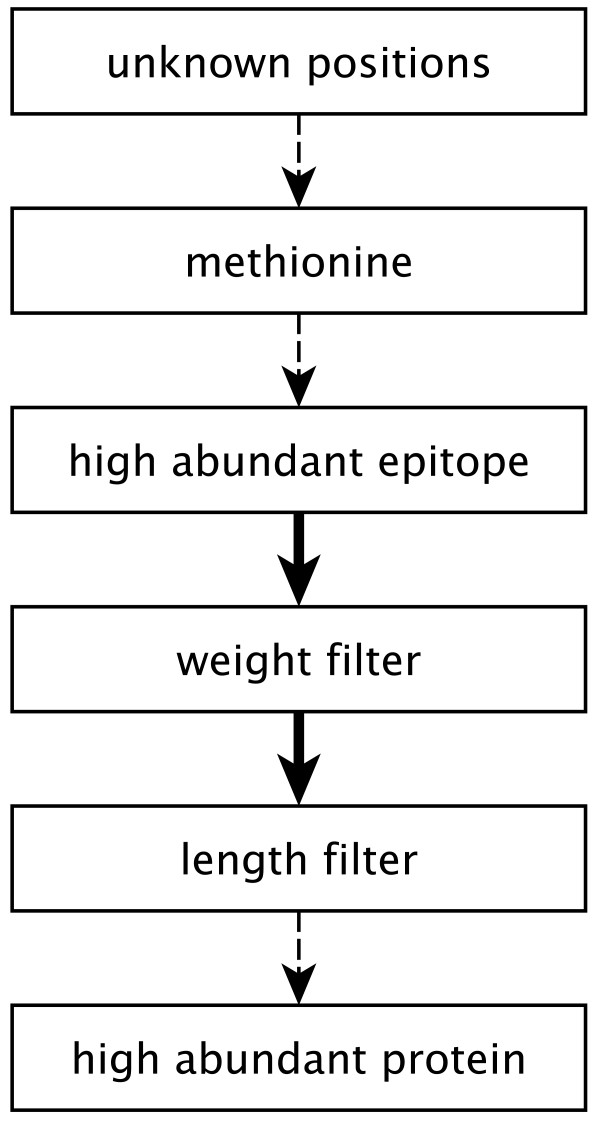
**Filter pipeline**.

Some proteins occur with great abundance in the sample, such as actin or tubulin. Terminal epitopes of peptides from these proteins are unsuitable as epitopes for immunoaffinity experiments for the same reasons explained in the high abundant epitope filter. In this last filter step an epitope stop list, generated from a hand cured list of high-abundant proteins, is used to remove those from list of combinations. As shown in figure 2 filters are usually applied in a specific order. While the methionine, unknown positions, high abundant protein filters can be applied at any position in the pipeline, other filters are order-dependent. This is the case if a filter evaluates the expected peptide distribution *C*^*e *^of an epitope *e*. These filters cannot be preceded by filters that change those distributions. The high abundant epitope filter must precede the weight filter, which must precede the length filter.

Through the application of this filter pipeline the preselection of epitopes is adjusted to the experimental setup and the problem dimension is significantly reduced.

The influence of the filters is shown in Table 1. While the unknown-positions-filter and the methionine-filter have a relatively small impact, the high abundant epitope filter and the weight filter remove a large number of combinations. The weight filters reduce the number of combinations by about 43%, while the number of epitopes is only reduced by 3%. The filter removes combinations from the set, which cannot contribute to the coverage (overlapping peaks). Still the corresponding antibody can capture peptides that are detectable by the mass spectrometer.

**Table 1:** Filter impact

Filter	# epitopes	# proteins	# combinations
Unfiltered	671,427	20,333	4,196,636

unknown positions filter	671,253	20,333	4,195,788

methionine filter	569,365	20,332	3,839,772

high abundant epitope filter	569,354	20,332	3,312,617

weight filter	559,323	20,178	1,962,034

length filter	530,863	20,020	1,662,437

high abundant protein filter	527,164	20,010	1,598,289

Impact of the different filters applied on the in-silico tryptic digest of the human proteome (Uniprot taxon id 9606), N-C-terminal epitopes of length 4 and 5, Δ_*min *_= 4,

Some antibodies ('robinson antibodies') capture only one peptide from one protein. If there is an antibody that captures more peptides from the same protein and others, it is always better to choose this one over the 'robinson antibody'. Therefore all robinson antibodies are removed from the graph before the optimization starts.

### Protein set cover problem formulation

The bipartite graph *G *= (*P *∪ *A*, *E*) is constructed by adding proteins and epitopes as vertices, and by connecting a protein node from the protein set *P *and an epitope node from the epitope set *A *if a combination appears in the filtered set:(5)(6)

The problem is to select a minimal set of antibodies *A*_*min *_⊂ *A *so that every protein in *P *is covered by at least one epitope. The minimum set cover is a classical problem in computer science and complexity theory.

The set cover can be formulated as a decision problem, where the question is asked, if a covering set of size k or less exists. This problem was shown to be NP-complete and achieving approximation ratios is no easier than computing optimal solutions. [12] The optimization version where the smallest covering set has to be found is NP-hard. It was shown that a greedy algorithm (see appendix) has an approximation ratio of(7)

where *n *is the size of the largest set. [13]

This the best approximation ratio for the set cover problem [14]. In this algorithm in each step the epitope in *A *covering the most yet uncovered proteins in *P*, is added to the solution set *L*, until all proteins are covered.

Another approach to the solution of the set cover problem is to formulate it as a binary linear program. The binary decision variables *s*_*a *_reflect the inclusion of an epitope *a *to the solution set. The number of the selected epitopes forms the objective function:(8)(9)(10)

The linear program is subject to the constraint that every protein P has to be covered by one or more epitopes in the solution:(11)(12)

This program can be solved with available solvers such as CPLEX or GLPK. This will lead to optimal solutions, if the problem dimension is small.

To enhance the accuracy of the proteomics experiments, it would be beneficial to capture the same or multiple peptides from a protein by different binders. In addition it is beneficial to include alternative binders in the experimental planning, in case the generation of a binder affine to a specific epitope fails. The multicovering problem (MCP) is a generalization of the set covering problem. Several algorithms have been proposed by Dobson [15], Hochbaum, Hall [16] and Rajagopalan [17]. Those heuristics would solve the problem of covering each protein twice or more. As it would be cost-prohibitive to double the number of binders, it is not possible to cover all target proteins more than once. This is the case at least for proteins that are covered by a very specific epitope. The following approach solves the pragmatic variant of the problem.

The greedy algorithm can be modified to enhance the probability of the selection of an epitope set that meets the multicoverage requirement for the target proteins.

In this variant (see appendix) the scoring function combines two different optimization targets, minimality and redundancy, by summation to a one-dimensional multiobjective fitness function.

The function is a weighted sum of the number of proteins which are not yet covered(13)

and the number of proteins which are covered again by this antibody(14)

*E *denotes the edge set in the bipartite graph and *P_cov _*the set of already covered proteins. The influence of new and already covered proteins on the overall score of an epitope is weighted by the parameters *s_mcov _*and *s_cov_*:(15)

Still the algorithm terminates with a total number of epitopes lower or equal as the number targets, because every added epitope is required to cover at least one new target protein.

The choice of the parameters *s_mcov _*and *s_cov _*has a high impact on the results, and depends heavily on the size of the dataset. The number of epitopes with high capacity is considerably lower in small datasets than in large datasets. Because of this the probability that a protein can be covered more than once by different high capacity epitopes is small. In large datasets the situation is the opposite. As many epitopes have a very large capacity, and possibly cover up to a few hundred peptides from many different proteins, it is more probable that the sets of captured proteins overlap. In this configuration it is better to score innovation over redundancy. While this is intuitively clear, it would be a big effort to determine the best values analytically. For large datasets should *s*_*mcov *_should be chosen smaller than *s*_*cov*_, for small datasets *s*_*mcov *_>*s*_*cov*_.

Multiple coverage can be integrated to the Integer Program formulation by changing the coverage constraints to(16)

for all proteins that can be covered twice. However this will lead to inclusion of elongated, already selected, epitopes (e.g. IER and EIER), to satisfy the double coverage constraints.

This formulation requires that all proteins are multiply covered by the solution. A better formulation reads as follows: Maximize the number of multiply covered proteins in a valid covering of all proteins, by using a fixed number of epitopes. The objective function maximizes the number of proteins which are multi-covered.(17)

If the binary variable *S*_*i *_is set to one, protein *i *has to be covered at least twice. This is guaranteed by using the following constraint:(18)

If *S*_*i *_is selected, at least two covering epitopes have to be selected in order to satisfy the constraint. This problem would be easily solved just by picking two epitopes randomly for each protein. In order the get an optimal usage of the epitopes their number is restricted by an additional constraint:(19)

Here *cost*_*max *_denotes the maximum number of antibodies to be chosen, and this has to be set by the user and may just depend on the available funding for antibody generation or purchase. An upper bound for *cost*_*max *_is the size of the optimal solution to the original multicover ILP, which already covers all proteins in the dataset twice or more. A lower bound is the minimal cost for the normal covering.

## Results and Discussion

Proteomes of various organisms (Homo sapiens, Mus musculus, Rattus norvergicus, Bos taurus, Saccharomyces cervisiae) were obtained from UniProtKB [18]. Only reviewed sequences were included in the dataset. The proteomes were trypsin-digested in-silico, by cutting after lysine (K) or arginine (R), if no proline (P) followed. A complete digest without missed cleavages or mis-cleavages was assumed. The resulting digests were pre-processed and filtered as described. To investigate the use case of assay designs for a limited number of targets, the lists of proteins associated to the pathways for TGF*ß *, WNT and TLR signaling were obtained from the KEGG (Kyoto Encyclopedia of Genes and Genomes) PATHWAY database [19]. The KEGG gene IDs in the pathway descriptions were mapped to Uniprot IDs. The combination sets for the pathways were extracted from the filtered combination set of the human proteome. The coverage score  of a solution *L *is the number of required epitopes relative to the number of proteins to cover.

The solutions of the integer program delivered by the industry standard ILP solver CPLEX after a limited running time of 12 hours were, not surprisingly, superior to the solutions provided by the greedy algorithm on all tested proteomes and epitope-length combinations (see Table 2). The inclusion of epitopes of length five increased the problem dimension considerably, because of the much larger number of potential epitope sequences (). This increased the number of coverable proteins in the final combination set.

Nevertheless the number of required epitopes was decreased in three of five proteomes (Homo sapiens, Rattus norvegicus, Bos taurus). When including terminal sequences of length four and five, the set cover will include shorter epitopes in most cases as they cover more proteins. If for a specific protein all epitopes of length four have been filtered out, longer sequences can still be used to cover it.

**Table 2:** Solutions for proteome datasets

Proteome	length	IP	Greedy	|A|	|P|
Homo sapiens	4-5	2,020 (10.1%)	2,292 (11.5%)	527,164	20,010

Mus musculus	4-5	1,541 (9.6%)	1,727 (10.8%)	473,406	15,995

Rattus norvegicus	4-5	851 (11.7%)	970 (13.3%)	273,558	7,295

Bos taurus	4-5	790 (14.2%)	903 (16.1%)	199,735	5,584

Saccharomyces cervisiae	4-5	1,000 (15.6%)	1,134 (17.6%)	240,253	6,422

Homo sapiens	4	2,026 (10.1%)	2,306 (11.5%)	86,963	19,979

Mus musculus	4	1,529 (9.6%)	1,737 (10.9%)	83,073	15,974

Rattus norvegicus	4	858 (11.8%)	975 (13.4%)	64,058	7,294

Bos taurus	4	792(14.2%)	896 (16.1%)	53,751	5,576

Saccharomyces cervisiae	4	995 (15.5%)	1,130 (17.6%)	58,464	6,405

Comparison of the solution quality of the integer program IP (CPLEX solver, running time limited to 12 hours) and the greedy set cover algorithm on proteomes of different species, and epitope length settings. |*A*| denotes the number of different epitopes, |*P*| the number of target proteins, the percentage next to solution sizes is the coverage score

The solutions provided by the multicoverage integer program are significantly larger than the solutions, in which multicoverage was not enforced. The multicoverage greedy approach only favors but does not enforce multicoverage, so the solutions provided by this method are smaller, but not necessarily superior to those provided by the multicoverage integer program. As shown in Table 3 the number of multicovered proteins (Homo sapiens, *length *= 4) was increased from 14,847 (Greedy) to 15,853 (Greedy MC) by only eight additional epitopes in the solution, compared to the solution of the standard greedy algorithm. This was achieved with a setting of *s*_*cov *_= 100; *s*_*mcov *_= 1, which scores not yet covered proteins one hundred times higher than already covered proteins. The solution of the IP MC is 3.895 large, so the effort of multicovering all 19,756 proteins nearly doubles the number of epitopes compared to the solution of the standard IP, where only 13,815 proteins are multicovered. By using IP MMC with the *cost*_*max *_set to the solution size of the Greedy MC the number of multicovered proteins was increased from 15,853 to 16,314.

**Table 3:** multicoverage of the human proteome

Solver	# prot. single covered	# prot. multicovered	|*L*|
IP MC	223	19,756	3,895

IP MMC (*cost*_*max *_= 2,314)	3,665	16,314	2,314

IP	6,164	13,815	2,026

Greedy MC	4,126	15,853	2,314

Greedy	5,132	14,847	2,306

Comparison of the solution quality of the IP MC, IP MMC, the greedy set cover algorithm, and the modified algorithm (Greedy MC, *s*_*cov *_= 100; *s*_*mcov *_= 1) on the in-silico tryptic digest of the human proteome (Uniprot taxon id 9606), N-C-terminal epitopes of length 4, |*L*| denotes the total size of the solution.

On the smaller pathway datasets it is possible to calculate the best possible solutions with CPLEX and GLPK in a very short amount of time (less than 2 seconds). Table 4 shows a comparison of the solution size and the multicoverage percentages on pathway datasets. On pathway datasets the solution sets are proportionally larger than on proteome datasets. This was expected, because the probability of shared terminal epitopes is smaller if the number of target proteins is reduced. Nevertheless coverage scores of 42% (WNT, length = 4-5, IP, 55 epitopes to cover 133 proteins) are a substantial improvement to the scenario of choosing peptide-or protein-specific antibodies for immunoaffinity-MS. The multicoverage integer program provided solutions with coverage scores from 81% (WNT, length = 4-5) to 100% (TLR, length = 4).

**Table 4:** Pathway results

Pathway	length	IP	IP MC	Greedy	Greedy MC	IP MMC	|P|
WNT	4-5	55 (13.5%)	107 (96.2%)	60 (22.6%)	88 (54.1%)	88 (77.4%)	133

TGF	4-5	36 (8.9%)	70 (93.7%)	40 (19.0%)	57 (49.4%)	57 (74.7%)	79

TLR	4-5	47 (5.3%)	92 (94.7%)	51 (16.0%)	72 (46.8%)	72 (70.2%)	94

WNT	4	56 (16.6%)	108 (94.0%)	63 (15.8%)	85 (48.8%)	85 (70.7%)	133

TGF	4	36 (8.9%)	71 (89.9%)	39 (13.9%)	57 (48.1%)	57 (69.6%)	79

TLR	4	47 (3.2%)	94 (93.6%)	50 (11.7%)	70 (45.7%)	70 (62.8%)	94

Comparison of the solution quality of the integer program (IP), integer multicover (IP MC), the greedy set cover (Greedy) and multicover (Greedy MC, *s*_*cov *_= 1; *s*_*mcov *_= 10 ), integer maximization multicover (IP MC, *cost*_*max *_was set to result of Greedy MC), algorithm on different pathways (subsets of the Homo sapiens proteome), and epitope length settings. The percentage in parentheses is the degree of multicoverage on the dataset, e.g. a value 50% means that half of the proteins are multiply covered.

The settings of the multicoverage greedy algorithm were changed to *s*_*cov *_= 1 and *s*_*mcov *_= 10, because the probability of multicoverage through one epitope is proportional to the size of the datasets. In this way the multicoverage score begins to take effect earlier during the iterative optimization. Table 5 contains results of a grid search on the parameters of the greedy MC algorithm applied to the WNT pathway example. The multicoverage enhancing effect shows only if already covered proteins are scored higher than new proteins.

**Table 5:** Grid search on the parameters *s*_*cov *_and *s*_*mcov*_

*s*_*cov*_/*s*_*mcov*_	1	2	3	4	5	6	7	8	9	10
1	73 (50)	84 (65)	86 (67)	85 (65)	85 (65)	85 (65)	85 (65)	85 (65)	85 (65)	85 (65)

2	63 (36)	73 (50)	79 (60)	84 (65)	86 (67)	86 (67)	86 (67)	85 (65)	85 (65)	85 (65)

3	61 (33)	64 (37)	73 (50)	79 (60)	80 (61)	84 (65)	86 (67)	86 (67)	86 (67)	86 (67)

4	61 (33)	63 (36)	64 (37)	73 (50)	79 (60)	79 (60)	80 (61)	84 (65)	86 (67)	86 (67)

5	61 (33)	61 (33)	64 (37)	64 (37)	73 (50)	79 (60)	79 (60)	80 (61)	80 (61)	84 (65)

6	61 (33)	61 (33)	63 (36)	64 (37)	64 (37)	73 (50)	79 (60)	79 (60)	79 (60)	80 (61)

7	61 (33)	61 (33)	61 (33)	64 (37)	64 (37)	64 (37)	73 (50)	79 (60)	79 (60)	79 (60)

8	61 (33)	61 (33)	61 (33)	63 (36)	64 (37)	64 (37)	64 (37)	73 (50)	79 (60)	79 (60)

9	61 (33)	61 (33)	61 (33)	61 (33)	64 (37)	64 (37)	64 (37)	64 (37)	73 (50)	79 (60)

10	61 (33)	61 (33)	61 (33)	61 (33)	63 (36)	64 (37)	64 (37)	64 (37)	64 (37)	73 (50)

Grid search on the parameters *s*_*cov *_and *s*_*mcov *_of the modified greedy set cover algorithm (Greedy MC) on the WNT pathway, N-C-terminal epitopes of length 4: *solution size *(*multicovered proteins*)

If *s*_*cov *_is chosen bigger than *s*_*mcov *_the multicoverage effect almost completely vanishes on small datasets. After the calculation of the greedy multicover the resulting cost (solution size) was used as the cost limit *cost*_*max *_for the maximization multicover (IP MMC) formulation. The results were significantly better multicoverage percentages for all datasets for the same costs (Table 4, compare columns Greedy MC and IP MMC).

Results, coverage reports and the used software package SCPSolver are available on the website

http://www.ra.cs.uni-tuebingen.de/software/scpsolver/txp.

## Conclusions

Starting from the real-world lab engineering task, we have shown that the problem of choosing a minimal set of epitopes is equivalent to the well-known set cover problem. In combination with a filter pipeline that eliminates unsuitable peptide-epitope combinations, we proposed different methods for the solution of the problem.

For small datasets (a few hundred proteins) it is possible to solve the problem to optimality with minimal computational effort using commercial or free solvers. Larger datasets, like full proteomes, require the use of heuristics, or respectively a running time limitation of the branch-and-bound search in the integer program solvers. Large sets of proteins can theoretically be covered by TXP-antibodies with a fraction (down to 9.57%, see Table 3) of the otherwise required peptide-specific antibodies for every protein. We further proposed methods to enforce (IP MC) or enhance (Greedy MC, IP MMC) the multiple coverage of a protein for a better experimental design.

The results presented in this paper were used to generate concrete lists of candidate epitopes, which are currently in production and evaluation in the NMI lab. While it is not yet clear whereif the TXP approach scales up to the proteome level, first results are promising.

In future work scheduling of binder generation, to get a broad coverage effect of interesting targets early, and other experimental setups, e.g. sandwich immunoassays based on TXP-antibodies, will lead to new interesting design optimization tasks on a higher level of complexity.

## Competing interests

TJ, OP, DS and MT are co-authors of the patent application WO2007/112927/A1 which describes the Triple X Proteomics approach. HP, JS, TJ, OP, DS, MT and AZ are co-authors of an International Patent Application PCT/EP2009/0001230 that will be published in August 2010 and is related to the epitope selection approach for TXP described in this paper.

## Authors' contributions

HP wrote the paper. JS implemented large parts of the filter pipeline. HP implemented the optimization routines and SCPSolver. OP, DS, MT an TJ provided the biological and biochemical input, including the problem. AZ supervised the work and corrected the paper. All authors read and approved the final manuscript.

## Appendix

**Input**: bipartite epitope-protein graph *G*(*P *∪ *A*, *E*)

**Output**: set of epitopes L

*P*_*cov *_= ∅;

*L *= ∅;

**while ***P*\*P*^*cov *^= ∅; **do**

   **foreach ***a *∈ *A*\*L ***do**

      *//calculate how many new proteins are covered by the epitope a score*

      *score*(*a*) = |{(*a*, *p*) ∈ *E*|*p *∉ *P*_*cov*_}|;

   **end**

   *//select the epitope a with the highest score*

   *a*_*s *_= arg max_*a *_*score*(*a*);

   *L *= *L *∪ {*a*_*s*_};

   *P*_*cov *_= *P*_*cov *_∪ {*p*|(*a*_*s*_, *p*) ∈ *E*};

   *//remove the covered proteins from the graph*

   *G *= *G*((*A *∪ *P*)\(*P*_*cov *_∪ *L*), *E*\{*(a*_*s*_, *P*) ∈ *E*});

end

**return ***L*

                  **Algorithm 1**: The greedy set cover algorithm

**Input**: bipartite epitope-protein graph *G*(*P *∪ *A*, *E*), *s*_*cov*_, *s*_*mcov*_

**Output**: set of epitopes L

*P*_*cov *_= ∅;

*L *= ∅;

**while ***P*\*P*_*cov *_≠ ∅; **do**

   **foreach ***a *∈ *A*\*L ***do**

      *//here the score favorizes the multiple coverage of proteins*

      

   **end**

   *a*_*s *_= arg max_*a *_*score*(*a*);

   *L *= *L *∪ {*a*_*s*_};

   *P*_*cov *_= *P*_*cov *_∪ {*p*|(*a*_*s*_, *p*) ∈ *E*};

   *E *= {(*a*, *p*) ∈ *E*|∃(*a*,  ∉ *P*_*cov*_) ∈ *E*}):

end

**return ***L*

         **Algorithm 2**: The multicoverage greedy set cover algorithm (Greedy MC)
